# Extension of Chronological Lifespan by Hexokinase Mutation in *Kluyveromyces lactis* Involves Increased Level of the Mitochondrial Chaperonin Hsp60

**DOI:** 10.1155/2012/946586

**Published:** 2012-05-17

**Authors:** Lisa Rizzetto, Elena Zanni, Daniela Uccelletti, Ileana Ferrero, Paola Goffrini

**Affiliations:** ^1^Department of Genetics, University of Parma, Parco Area delle Scienze 11/A, 43100 Parma, Italy; ^2^Pharmacology Department, University of Florence, Viale Pieraccini 6, 50139 Firenze, Italy; ^3^Department of Biology and Biotechnology “Charles Darwin”, Sapienza University of Rome, P.le A. Moro 5, 00185 Rome, Italy

## Abstract

Oxidative damage, mitochondrial dysfunction, genomic instability, and telomere shortening represent all molecular processes proposed as causal factors in aging. Lifespan can be increased by metabolism through an influence on such processes. Glucose reduction extends chronological lifespan (CLS) of *Saccharomyces cerevisiae* through metabolic adaptation to respiration. To answer the question if the reduced CLS could be ascribed to glucose *per se* or to glucose repression of respiratory enzymes, we used the *Kluyveromyces lactis* yeast, where glucose repression does not affect the respiratory function. We identified the unique hexokinase, encoded by *RAG5* gene, as an important player in influencing yeast lifespan by modulating mitochondrial functionality and the level of the mitochondrial chaperonin Hsp60. In this context, this hexokinase might have a regulatory role in the influence of CLS, shedding new light on the complex regulation played by hexokinases.

## 1. Introduction

All living organisms undergo physiologic decline by aging. Several studies attempted to comprehensively characterize the environmental and genetic determinants of chronological lifespan (CLS). CLS is defined as the length of time stationary phase yeast cells remains viable in a quiescent state and it is generally determined by monitoring viability over time [1, 2]. Caloric restriction (CR), or the reduction of caloric intake without lack of essential nutrients, is capable of extending lifespan in a variety of laboratory animals, ranging from *Caenorhabditis elegans* to mice [3–7]. Yeast has proven to be a useful model organism for studying the genetic modulation of aging [8–10]. In *Saccharomyces cerevisiae*, the growth in rich media with low glucose concentrations significantly increases chronological lifespan (CLS) [3, 4]. *S. cerevisiae* is a Crabtree-positive yeast, in which fermentation is the preferred metabolic pathway and aerobic metabolism is inhibited, despite the presence of oxygen, when glucose levels are high [11]. Any mechanism that blocks the uptake and metabolism of glucose has the potential to act as CR mimic. Recently, in an effort to investigate if glucose repression [12, 13] is involved in aging regulation, it has been demonstrated that aerobic catabolism was necessary to increase longevity in *S. cerevisiae* [14]. In *Kluyveromyces lactis*, a Crabtree-negative yeast [12], glucose limitation does not promote an enhancement of respiratory capacity [15] nor a decrease in reactive oxygen species formation in conditions of CR as occurs in *S. cerevisiae *[14, 16, 17]. In addition, *K. lactis* does not exhibit an increased longevity when incubated in low glucose concentrations [14].

To answer the question whether the reduced CLS due to the presence of glucose could be ascribed to glucose *per se* or to glucose repression, we used *K. lactis, *since glucose repression does not affect the respiratory function. If such a repression was the source of cell lethality, glucose repression mutants would display an increased lifespan compared to the parental strain. We report here that only the absence of the unique hexokinase, encoded by *RAG5* gene, extends CLS in a CR-independent manner, indicating a specific role of this protein in reducing lifespan independently of glucose repression. The hexokinase defective mutant showed an increased lifespan, associated with mitochondrial membrane potential maintenance and increased transcriptional and translational levels of the mitochondrial chaperonin Hsp60. These findings suggest a putative regulatory role of hexokinase in influencing mitochondrial functions and CLS.

## 2. Material and Methods

### 2.1. Yeast Strains, Media, and Growth Conditions

 Wild type JA6 (*MAT*α* ade*1 *ade*2 *trp*1-2 *ura*3, kindly provided by K. Breunig) *K. lactis *and its isogenic mutant strains *rag1Δ* [18], *rag5Δ* [19], *rag4Δ* [20], and *mig1Δ* [21], were used in this work. All experiments in liquid medium were performed in complete medium (YP) with 2% glucose. For survival experiments, overnight cultures grown in YPD medium were used to inoculate experimental cultures on day 0 at a density of 1 × 10^5^ cells/mL into 500 mL flasks with a flask volume : medium volume ratio of 5 : 1. The growth was carried out at 28°C, shaking at 220 rpm. Viability was measured over time by plating serial dilutions of the cultures onto YPD plates (1% yeast extract, 1% peptone, 2% glucose, 2% agar) and counting colony forming units (CFU) after three days of growth.

### 2.2. Stress Resistance Assay

The cells grown until the early stationary phase were diluted to an OD_600_ of 0.2 and then incubated for two hours with or without menadione at the concentrations of 5 and 20 *μ*M. After the treatment about 100 cells were plated onto YPD plates and the colonies were counted after three days of growth at 28°C.

### 2.3. Measurement of Intracellular Oxidation Levels

The oxidant-sensitive probe dihydrorhodamine 123 (DHR123 (molecular probes)) was used to measure intracellular oxidation levels in yeast according to Zanni et al. [22], with slight modifications. Aliquots of cell cultures, challenged with menadione, were diluted to a final OD_600_ of 0.2. After the DHR 2 h of incubation, cells were concentrated by centrifugation and resuspended in 200 *μ*L of fresh medium. 2 *μ*L of the suspension were loaded onto slides and observed immediately under fluorescence microscopy (excitation at 488 nm and emission at 530 nm). At least 600 cells per sample were scored manually as fluorescent or nonfluorescent.

Total glutathione content was measured by adapting GSH/GSSG-Glo Assay (Promega, Italy) with slight modifications. Aliquots of cell cultures were taken at the time indicated. Protein extraction was performed in lysis buffer, supplied by the kit, trough mechanical disruption using glass beads. Following reactions performed upon manufacturers' indications, stable luciferin luminescent signals were detected using a GloMax luminometer (Promega, Italy). The luminescent signals, proportional to the amount of GSH, were then correlated with the total protein content, measured by using Bradford assay. The results are expressed in nM of GSH per mg of total protein.

### 2.4. Fluorescence Microscopy

Cells grown in YPD medium were harvested at different time-points, washed with water, and then incubated with the different dyes as follows. For mitochondrial functionality analysis the vital dye 2-(4-dimetylaminostyryl)-*N*-methylpyridinium iodide (DASPMI) was used as previously described [23].

For mitochondrial nucleoids visualization, cells were vitally stained with 4′,6-diamidino-2-phenylindole (DAPI) in PBS at a final concentration of 0.6 *μ*g/mL for 20 min [24]. Epifluorescence microscopy was carried out with a Zeiss AxioVert 25 microscope fitted with a 100x immersion objective and standard FITC (DASPMI) or UV (DAPI) filter sets.

### 2.5. Flow Cytometry

Flow cytometry was performed on Accuri C6 Flow Cytometer. Analysis of yeast ROS using dihydroethidium (DHE) was performed as previously described [25]. Samples were taken from the indicated cultures and cells were pelleted by centrifugation. After the removal of the media, the cell pellets were resuspended in phosphate-buffered saline (PBS) containing 50 *μ*M DHE and incubated at 30°C for 10 minutes. Cells were then pelleted and resuspended in PBS without dye. The DHE fluorescence indicated is the direct output of the FL3 (red-fluorescent detecting) channel.

Analysis of yeast ROS using DHR123 was performed as previously indicated [26]. Briefly, at the time indicated, cells were pelleted by centrifugation and washed with PBS. DHR123 (molecular probes) was added at 50 *μ*M. Cells were then stained for 10 minutes at 30°C, washed twice with PBS, and analyzed by flow cytometry using the FL1 channel.

Measurement of mitochondrial potential was performed as described by Pan and Shadel [27]. Briefly, at the time indicated, cells were pelleted by centrifugation and washed with PBS. DiOC_6_ (molecular probes) was diluted to a final concentration of 200 nM in PBS, and used to resuspend the cells. The cells was then incubated for 30 minutes at 30°C, washed twice with PBS and analyzed by flow cytometry using the FL3 channel.

For each assay, a counter staining was performed using 1 mg/mL propidium iodide (PI) to discriminate between live and dead cells. Dual compensation was performed to remove signal bleed between the PI and FL1 or FL3 channel. A total of 50000 PI-negative gated cells was analyzed for each curve. For each measurement, the mean fluorescent intensity is plotted ± standard deviation.

### 2.6. Northern Blot Analysis

Total RNA was prepared by hot acidic phenol extraction [28] from cells grown in YP supplemented with 2% glucose and harvested after 24 hr and 48 hr, 4, 7, and 10 days. Northern analysis was carried out according to a previously established protocol [29]. The probes were obtained by PCR amplification using genomic DNA of *K. lactis* strain 2359-152 as template. The primers used were the following: SOD1 Fw: 5′-AGTTGCAGTTTTGAAGGGTGA-3′, SOD1 Rv: 5′-TACCAATAACACCACAGGCGT-3′; SOD2 Fw: 5′-TGTAGCCCGTAGAGCTTTGCA-3′, SOD2 Rv: 5′-AGCAGCAGCTTCAAATCTCTT-3′; CTA1 Fw: 5′-CGGACGTGTGCGGTTCAGCGAT-3′, CTA1 Rv: 5′-CAGCAGAAGCTTCTTGGTATGG-3′; CTT1 Fw: 5′-GTCTGACATGAGTGCATATTCCG-3′, CTT1 Rv: 5′-CGACTGGAGATGCACCATTGAC-3′; 26S rRNA Fw: 5′-GCCAGCTGGGACTGAGGATTGC-3′, 26S rRNA Rv: 5′-CCCAAAGCCTTTCCGCTCCGAAGC-3′. *KlHSP60* probe corresponded to the 1722 bp region *Eco*RI-*Eco*RI derived from the plasmid p426-ADH-SD11 [30]. The amount of RNA loaded on the gel was estimated using hybridization with a *K. lactis* 26S probe. Probes were labelled with (a32P) dCTP by the Rediprime DNA labelling system (Amersham).

### 2.7. Western Blot Analysis

Total cell extract was obtained by breaking the cell wall with glass beads. The cell extract was mixed with 2 *μ*L of sample buffer (0.2 M Tris-HCl (pH 6.8), 30% glycerol, 1.2% sodium dodecyl sulfate (SDS), 30% *β*-mercaptoethanol, and 0.05% bromophenol blue). After 3 min at 95°C, the solution was resolved by 15% SDS-polyacrylamide gel electrophoresis (PAGE). After gel electrophoresis, the amounts of total protein were evaluated by densitometry after Coomassie blue R-250.

For western analysis, after electrophoresis, the gel was electroblotted onto a polyvinylidene difluoride membrane (Bio-Rad, Hercules, CA, USA) in Towbin buffer at 200 V for about 1 hour. Primary polyclonal antibodies were used at a dilution of 1 : 2000 (Stress-Gen, Victoria, British Columbia, Canada). The secondary antibody was goat anti-rabbit conjugated with peroxidase (Bio-Rad, Hercules, CA, USA). A detection kit (PIERCE) was used according to the manufacturer's instructions.

### 2.8. DNA Manipulation

 Published procedures were used for the transformation of *K. lactis* [31] and *Escherichia coli* [32] and for the isolation and purifications of plasmids from *E. coli*, agarose, and gel electrophoresis [33]. *E. coli DH10B *(*mcr*A *Δ*(*mrr-hsd* RMS-*mcr* BC) Φ80dD *lac*Z*Δ*M15*Δlac*X 74 *deo*R *rec*A1 *end*A1 *ara*D139 *Δ*(*ara, leu*)*7697 gal*U *gal*k*λ*-*rps*L*hup*G) was used for plasmid propagation and maintenance.

### 2.9. Cytochrome Spectra and Respiration

Cytochrome spectra and respiration were determined as described previously [34]. Oxygen uptake was measured at 30°C using a Clark-type oxygen electrode in a 1 mL stirred chamber containing 1 mL of air-saturated respiration buffer (0.1 M-phthalate-KOH, pH 5.0), 10 mM glucose (Oxygraph System Hansatech Instruments England) starting the reaction with the addition of 20 mg of wet weight of cells as previously described.

## 3. Results and Discussion

### 3.1. Evaluation of Chronological Lifespan in Glucose Repression Mutants and Identification of *rag5Δ* Long-Lived Strain

In *S. cerevisiae*, previous hypotheses state that high glucose concentration reduces cell viability causing a repression of respiration and that glucose restriction increases longevity by raising respiration level [35]. We first investigated whether glucose caused a decrease of CLS by accelerating yeast cell death in *K. lactis*, species in which glucose does not repress the respiratory pathway. This characteristic appears quite important since it allows the evaluation of the response to glucose in terms of cell longevity independently of the effect of glucose on respiratory activity.

**Figure 1:**
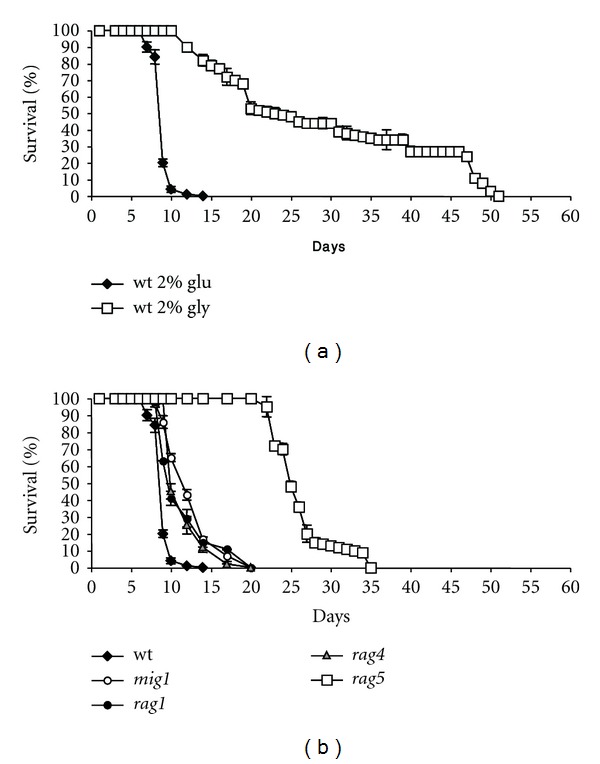
Chronological lifespan of yeast *K. lactis*. (a) Comparison of chronological survival of wild type JA6 grown in either glucose 2% and glycerol 2%. (b) Comparison of chronological survival in YPD medium of wild type strain and several mutants in glucose repression pathway. For each analysis (a, b) aliquots of cultures were counting over time and serial dilutions were plated on solid medium. Colony forming units were counted after 3 days. Data are representative of at least three independent experiments, mean ± s.d.

Since it has been observed that growth curves generated from cells in synthetic complete (SC) minimal medium are more variable [36], we decided to assess CLS in complete medium (YP). Therefore we analyzed the survival rate in cells grown in medium containing glucose (2% YPD) or nonfermentable carbon source such as glycerol (2% YPG) (Figure 1(a)). The results indicated that, as previously observed in *S. cerevisiae*, *K. lactis* cells grown in glucose died more rapidly than cells grown in glycerol although respiration levels were similar (data not shown). 

To answer the question if glucose cell death was due to the general regulation exerted by glucose, namely glucose repression, we measured the lifespan of four nonglucose repressible mutants: *rag1Δ* (*RAG1* encodes for a glucose-low affinity transporter) [37, 38]; *rag4Δ* (*RAG4* encodes for the unique glucose sensor) [20], *mig1Δ *(*KlMIG1* presents structural and functional homology with its orthologous in *S. cerevisiae, *encoding a protein involved in transcriptional repression) [21] and *rag5Δ* (*RAG5 *gene encodes for the unique hexokinase) [39] (Figure 1(b)). These data indicated that the lifespan reduction observed in glucose medium was not due to the repression *per se,* since the nonrepressible mutants displayed a survival rate similar to wild type. As only exception, *rag5Δ* mutant showed a survival significantly higher than the other strains (Figure 1(b)).

This result demonstrated that the reduced cell viability observed in glucose compared to glycerol was not due to glucose repression. Thus a role of *RAG5* gene, in glucose cell death, should be instead considered.

**Figure 2:**
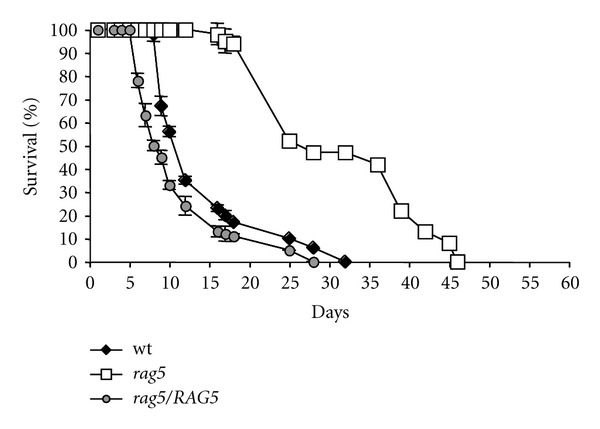
Evaluation of gene dosage of *RAG5* in determining the lifespan. Comparison of chronological survival in YNB medium of wt and mutant lacking *RAG5* and the same strain overexpressing *RAG5*. Survival was assessed in minimal medium for plasmid maintenance. Over time, aliquots of cultures were counted and serial dilutions were plated on solid medium. Colony forming units were counted after 3 days. Data are representative of five independent experiments, mean ± s.d.

To test if the extended lifespan was not due to a possible genetic rearrangement during gene disruption, we reintroduced *RAG5* gene on a plasmid into the *rag5Δ* mutant strain and determined the CLS. The expression of RAG5 completely restored the parental phenotype in mutant cells (Figure 2). To maintain the recombinant plasmid the analysis was carried out on minimal mineral medium (YNB). As expected, the growth in YNB caused an increase in CLS both in mutant and wild type cells due to the reduction in nitrogen content, as previously described in *S. cerevisiae* [3, 4].

A mutation in *HXK2* gene, which encodes one of the three sugar kinases of *S. cerevisiae*, has been reported to be associated with increased lifespan. This genetic model mimics calorie restriction by limiting glucose availability inside the cell [4]. However, in *K. lactis* we observed that the absence of the glucose-low affinity transporter Rag1p did not cause an increase of cell viability despite the fact that the mutation reduces glucose inside the cells [37]. This strongly suggested that the beneficial effect of *rag5Δ* mutation should not be attributed to the reduced glucose availability.

### 3.2. Oxidative Stress during Chronological Aging in the *rag5Δ* Long-Lived Strain

**Figure 3:**
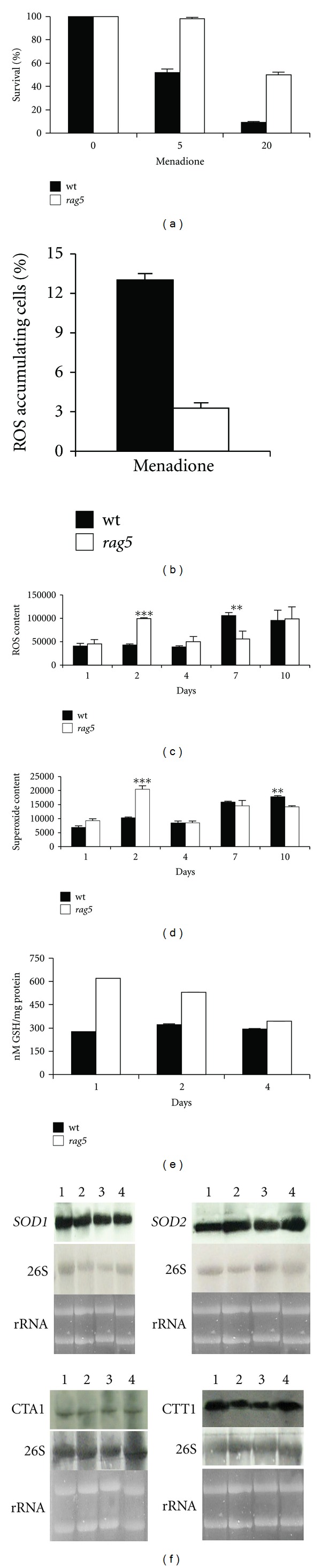
Oxidative stress analysis in *rag5Δ* strain. (a) Evaluation of stress resistance of the long-lived strain by inducing ROS damage with 5 and 20 *μ*M menadione. (b) Evaluation of ROS accumulation in 1 day cultures of wt and *rag5* cells challenged with 20 *μ*M menadione for 2 hours. The results were the mean of at least three independent experiments with s.d. <5%. (c, d) Measurement of cellular ROS. FACS analysis of aging cells stained for cellular ROS using DHR123 (c) and cellular superoxide using DHE (d) are shown. The mean fluorescent intensity of three independent experiments is plotted ± one standard deviation.**P value from a Student's *t*-test, <0.01, ***P value from a student's *t*-test, <0.001. (e) Total glutathione levels. GSH levels were normalized with respect to the protein total content of the samples. The results are the mean of three independent experiments with s.d. <5%. (f) Northern analysis of *CTA1*, *CTT1*, *SOD1,* and *SOD2* in wt strain and in *rag5Δ* strain. Total mRNAs were prepared as described in Section 2 from cells grown on YPD medium for 24 and 48 hours. Lines 1: wt, 24 h; line 2: *rag5Δ, *24 h; lines 3: wt, 48 h; line 4: *rag5Δ*, 48 h. The experiment showed is representative of three independent experiments, repeated with similar results.

 Oxidative modifications of cellular components, through the action of reactive oxygen species (ROS), have been described as one of the main contributions to an aged phenotype [8, 17, 40]. To evaluate if the aging delay observed in cells lacking the hexokinase was associated with a major resistance to ROS, the effect of menadione, a well-known oxidative stress generator [41], was analyzed. The lack of *RAG5* gene resulted in increased resistance to the stress induced by this chemical agent. In fact, approximately 50% of *rag5Δ* cells were viable after exposure to the highest dose (20 *μ*M), whereas only 9% of wild type cells survived to the treatment (Figure 3(a)). In addition, while wild type cells challenged with menadione showed a high increase of ROS, mutant cells showed a 4-fold decreased amount of ROS respect to wild type counterpart as visualized by dihydrorhodamine 123 (DHR123) staining (Figure 3(b)).

These results prompted us to investigate the intracellular oxidation levels along the CLS by using the same approach. Wild type cells showed a slight increase in ROS level starting from the 4th day of growth (data not shown). On the other hand, the long-living mutant displayed a huge accumulation of ROS after only 2 days. Surprisingly these intracellular oxidation levels decreased on the 4th day, becoming similar to those of wild type strain only after 10 days of incubation (data not shown). Analysis of cellular ROS by FACS further confirmed the different ROS accumulation between the two strains, highlighting even more the huge pulse of ROS in the mutant cells at 2 days of growth (Figure 3(c)). Analysis of cellular superoxide using DHE staining indicated the involvement of mitochondria in determining the observed ROS accumulation (Figure 3(d)).

Cells counteract oxidative damage activating the synthesis of a number of protective enzymes and molecules, such as glutathione (GSH) and detoxifying enzymes. Our data led to consider whether *rag5 *mutation caused an increase in GSH content as well as a different expression of genes involved in the oxidative stress response. In particular we observed that mutant cells showed an increased GSH content already at 1 day of growth (Figure 3(e)).

Then, we analyzed the transcriptional levels of *SOD1 *and* SOD2* genes, encoding the cytosolic Cu, Zn-superoxide dismutase and the mitochondrial Mn-superoxide dismutase, respectively. The *CTA1* and *CTT1* transcripts, coding for the cytosolic and mitochondrial isoforms of catalase, respectively, were also analyzed (Figure 3(f)). No significant differences were observed, suggesting that neither the increased resistance to menadione nor the ongoing oxidative stress, taking place in the mutant strain, seems to be accomplished by an increase of defence enzymes, although there was an increased level of GSH.

### 3.3. Mitochondrial Functionality in the *Rag5Δ* Long-Lived Strain

**Figure 4:**
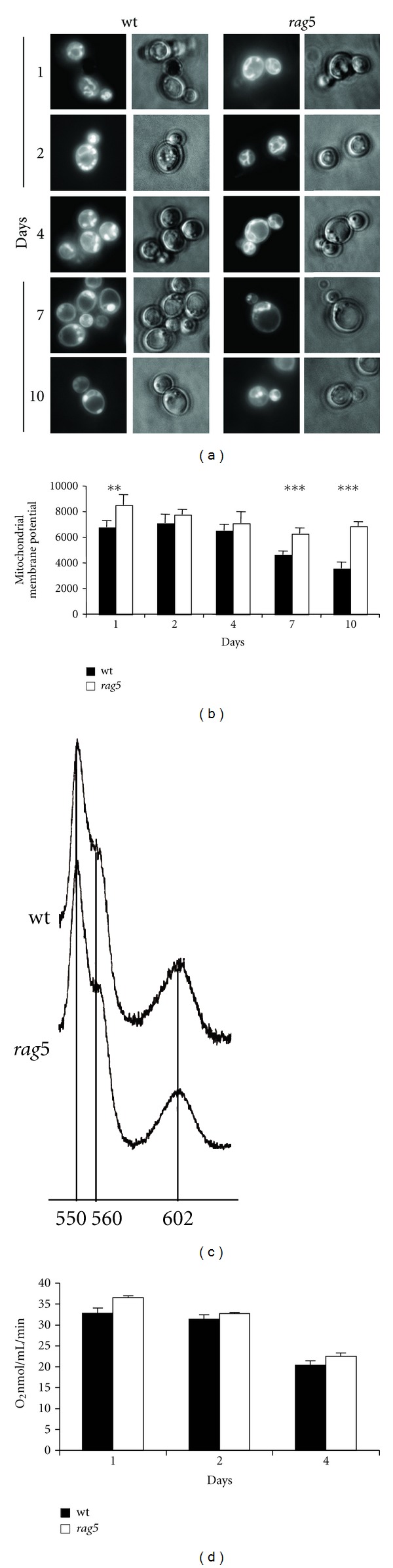
Functional analysis of mitochondria in the long-lived mutant. (a) Comparison of mitochondrial morphology of *rag5Δ* and wt strains after growth in YPD medium. Analysis was performed by taking aliquots at the indicated times and observing cells after DASPMI staining at the fluorescence microscopy. (b) Mitochondrial membrane potential determined by DiOC_6_ staining and FACS analysis. Values are expressed as mean fluorescent intensity. Means of four biological replicates ± one standard deviation are graphed. **P value from a Student's *t*-test, <0.01, ***P value from a Student's *t*-test, <0.001. (c) Cytochrome spectra. Cell samples were collected at day 1 and cytochrome spectra were evaluated. The peaks at 550, 560, and 602 nm (vertical bars) correspond to cytochromes c, b, and aa3, respectively. The height of each peak relative to the baseline of each spectrum is an index of cytochrome content. Data are representative of three independent experiments, repeated with similar results. (d) Oxygen consumption rates in wt and *rag5Δ* cells. Cell samples were collected at days 1, 2, and 4 and oxygen consumption was measured at 30°C using a Clark type oxygen electrode. Data are means ± s.d. of three independent experiments. Data were normalized for the number of live cells.

Since mitochondrial damage, exerted by ROS accumulation, represents one of the most important aging features, we focused our analysis on mitochondrial changes along the CLS. Nowadays controversial findings have been reported on the role of ROS and mitochondrial respiration in aging. Several studies addressed the positive relationship between increased mitochondrial metabolism and extension of lifespan [9, 16, 27]. In conflict with Harman's free radical theory of aging, these effects may be due to increased formation of ROS within the mitochondria. As recently reported in *S. cerevisiae*, in long-lived mutants this metabolic alteration is accomplished by increased respiration and increased mitochondrial membrane potential [42]. Thus, we wondered if the hexokinase activity, in *K. lactis *cells, could play a negative role in mitochondrial maintaining during age. To this aim, cells were incubated with DASPMI, a fluorescent probe that is taken up by mitochondria as a function of membrane electrochemical potential. We found indeed a different distribution of the dye between the two strains (Figure 4(a)). Specifically, in the wild type background, cells showed a punctuate pattern representing the typical tubulation phenotype. This feature was already visible after 2 days of growth, becoming ever more evident during CLS. By contrast the *rag5Δ *mutant showed a lasting maintenance of the tubular mitochondrial network resulting in a significant delay in the appearance of the tubulation pattern: 7 days compared to the 2 days of the wild type counterpart.

In *S. cerevisiae *a marker of aging is the mitochondrial membrane potential (MMP) decline [43]. To further evaluate if the *rag5* mutation induces this phenotype, the MMP was measured using FACS [27]. DiOC_6_ staining indicated that also in *K. lactis* MMP declines with age. On the contrary, *rag5* mutant cells maintain the MMP over time (Figure 4(b)).

Since the respiratory capability is a relevant player in the cellular redox balance, we compared the oxygen consumption of wild type and long-lived strains at different time-points. Moreover, mitochondrial respiratory structures were also investigated. In both analyses no significant differences were found between the two strains (Figures 4(c) and 4(d)).

Thus, the observed accumulation of oxidative stress in *rag5Δ* mutant could be necessary to delay the aging process and ameliorate mitochondrial function as recently proposed [42, 44].

In recent time, the role of MMP in determining the lifespan of *S. cerevisiae* has also been addressed [45]. The deletion of the mitochondrial protein translation factor *MEF2* gene has been shown to decrease longevity altering the mitochondrial membrane potential.

### 3.4. Is Hsp60p Involved in Chronological Lifespan?

**Figure 5:**
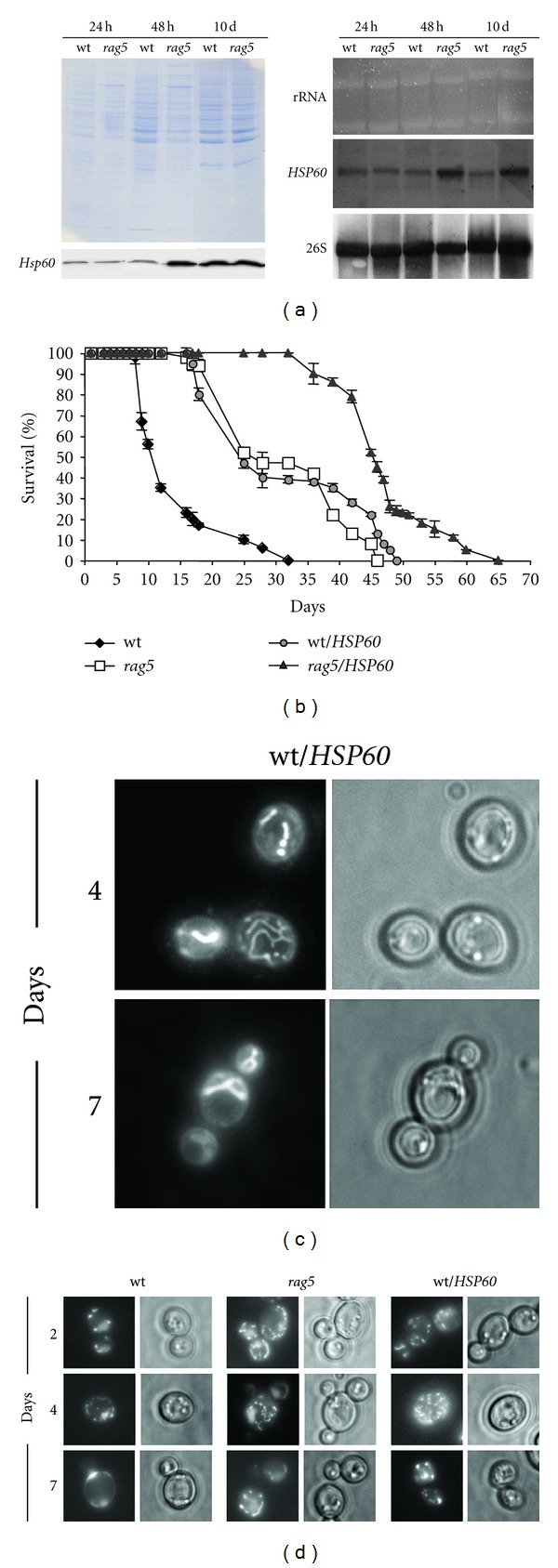
Analysis of involvement of Hsp60 in chronological aging and mitochondrial functionality. (a) Analysis by SDS-PAGE, western blotting of Hsp60 protein and northern analysis of *HSP60 *in the wt and in the isogenic *rag5Δ* strain. Determination of protein level by western analysis was performed as described in Section 2. Total mRNAs were prepared as described in Section 2 from cells grown on YPD medium for 24, 48 hr, 10 days. RNA was hybridized with labelled probes for *HSP60* and 26S. Data are representative of three independent experiments, repeated with similar results. (b) Evaluation of gene dosage of *HSP60* in determining the lifespan. Comparison of chronological survival in YPD medium of wt, *rag5Δ* strain, and the same strains overexpressing *HSP60* gene. Survival was assessed in minimal medium for plasmid maintenance. Over time, aliquots of cultures were counted and serial dilutions were plated on solid medium. Colony forming units were counted after 3 days. Data are representative of three independent experiments, mean ± s.d. (c) DASPMI staining in wt cells overexpressing *HSP60* gene after growth in YPD medium. Analysis was performed by taking aliquots at the indicated times and observing cells after DASPMI staining at the fluorescence microscopy. (d) Evaluation of mitochondrial nucleoids morphology along CLS in *rag5Δ*, wt and wt/HSP60 strains. Samples were collected at the indicated time-points and fluorescence was observed after DAPI *in vivo *staining.

According to recent reports, enhanced expression of the essential mitochondrial chaperone Hsp60 in yeast cells led to increased resistance to oxidative damage [46], process known to affect chronological-aged yeast cells [47]. Moreover, in *S. cerevisiae* Hsp60 has a role in the stability and transmission of mtDNA nucleoids [48]. These are foci for mtDNA replication and transcription, and are essential for cells to maintain respiratory competence as well as mitochondrial biogenesis [49]. These findings prompted us to evaluate Hsp60 expression in *rag5Δ *strain. Western blotting analysis showed that the highest protein amount was achieved in *rag5Δ* cells after 48 h, declining afterward to level similar to that of the *RAG5 *parental strain (Figure 5(a)). Northern blotting analysis overlaps the results obtained by western blotting, indicating that the increased protein amount was due to an enhanced gene transcription (Figure 5(a)).

Our next working step was to speculate on a possible role for hexokinase in the oxidative stress resistance by modulating Hsp60 expression. To this aim, *rag5Δ *and wild type strains were transformed with a multicopy vector carrying the *HSP60* gene and CLS was evaluated in both transformed strains. *HSP60* overexpression in *K. lactis* cells resulted in a lifespan extension similar to that of *rag5Δ *strain transformed with the empty vector (Figure 5(b)). Moreover, increased dosage of this mitochondrial chaperone gene in the hexokinase null mutant further enhanced its longevity.

Since in the mutant cells a delay in mitochondrial alteration was observed, we wondered if this condition was Hsp60-dependent. To this aim we analyzed the mitochondrial functionality of the wild type strain overexpressing *HSP60 *gene. Notably DASPMI staining of these cells completely resembled that of *rag5Δ;* in fact, both the mitochondrial morphology and the tubulation pattern appearance were almost identical to those of the mutant cells (Figure 5(c)).

In *S. cerevisiae *it has been reported a role for Hsp60 in nucleoids remodelling under metabolic cues [50]. To reinforce the link between the mitochondrial chaperone and the hexokinase, the mitochondrial DNA morphology was evaluated by DAPI staining (Figure 5(d)). While *rag5Δ *strain maintained mitochondrial integrity over the 7th day of growth, mitochondrial nucleoids disappeared in wild type cells after only 4 days. Moreover, *HSP60* overexpression in wild type cells was sufficient to extend mito-nucleoids maintenance.

Recently, an increased level of Hsp60 has been observed in two long-lived mutant of* Podospora anserina* associated with a retrograde response activation [51]. In *K. lactis*, similarly to this filamentous fungus, the hallmark of aging could be the reorganization of the mitochondrial DNA (mtDNA) accompanied by mitochondrial dysfunction. Our observation suggests a negative role of *RAG5* gene in maintenance of mitochondrial health.

## 4. Conclusion

A role of metabolism in determining aging has been reported in several organisms. In *K. lactis*, similarly to the aging yeast model *S. cerevisiae*, glucose caused a reduced viability compared to glycerol and, contrary to *S. cerevisiae*, calorie restriction (glycerol) increased longevity without raising respiration. Our results suggested a role of *RAG5* gene in glucose cell death, different from its role in glucose repression.

We showed that the long-lived mutant was more resistant to oxidative stress, coupled by increased levels of *HSP60*. Furthermore, an increase in copy number of *HSP60* extended lifespan at the same level as *rag5Δ *mutation, suggesting a negative control of *HSP60* by Rag5p.

The increased longevity in *rag5Δ* mutant was associated with a delay in mitochondrial dysfunction associated to membrane potential maintenance, stressing the role of mitochondria in promoting longevity as previously reported (for a review see [8]). Here, we provide evidence that the lack of the unique hexokinase and the accumulation of ROS during growth are adaptive mitochondrial signals that program a delay in mitochondrial membrane potential decline and a decrease of oxidative stress in aged cells, promoting longevity in a process recently known as mitochondrial hormesis or mitohormesis [44].

In this view, the aging process is an active process in which Rag5p plays an important role mainly by negative regulation of mitochondrial functionality and Hsp60 activity.

Further investigations are needed to explore the molecular mechanism underlying this process, but our study opens new horizon in regulative role of hexokinase.
